# Comparison of Efficacy of Ivabradine With Traditional Therapy in Patients With Left Ventricular Dysfunction

**DOI:** 10.7759/cureus.19192

**Published:** 2021-11-01

**Authors:** Umair Ali, Tanveer Ahmad, Jehanzeb Khan, Muhammad ijaz Khan, Hurriya Khan, Bilal Javed

**Affiliations:** 1 Cardiology, Khyber Teaching Hospital Peshawar Pakistan, Peshawar, PAK; 2 Cardiology Department, Qazi Hussain Ahmed Medical Complex, Nowshera, PAK; 3 Cardiology, Lady Reading Hospital, Peshawar, PAK; 4 Oncology, Sheffield Teaching Hospitals NHS Foundation Trust, London, GBR; 5 Medicine Unit, Khyber Teaching Hospital, Peshawar, PAK; 6 Internal Medicine, University Hospital, Tralee, IRL; 7 Medicine, Khyber Medical College, Peshawar, PAK; 8 Medicine, Quaideazam Medical College, Bahawalpur, PAK

**Keywords:** traditional therapy, re-admissions, nyha class, congestive heart failure, ivabradine

## Abstract

Background: Heart failure patients usually present with disease exacerbation that overburdens the hospitals and also increases the risk of mortality with increased heart rate being the main issue. Consideration is being given to drugs for sole heart rate control in addition to conventional therapy.

Objective: To compare the outcomes of ivabradine to traditional treatment in patients with left ventricular systolic dysfunction.

Methodology: This randomized controlled trial was conducted in the Department of Cardiology, Khyber Teaching Hospital, Peshawar from November 1, 2020, to May 31, 2021. Patients aged 30-65 years of age and of either gender with heart failure were enrolled in the study. Patients were screened for New York Heart Association (NYHA) class and were enrolled into one of the two groups. In group 1, patients were started on traditional treatment, while group 2 patients were given ivabradine as an add-on therapy. Follow-up was made at the end of the second month for evaluation of the outcomes.

Results: Each group had 119 patients, with a mean age of 58.05±4.98 years. Group 1, consisted of 61.3% of the patients in NYHA 3, while 38.65% were in NYHA 4. In group 2, NYHA 3 and NYHA 4 patients were 59.6% and 40.3%, respectively. Upon follow-up, there were greater improvements in group 2 as compared to group 1 based on NYHA classifications, with NYHA 2 [47.05% (group 2) vs. 13.44% (group 1)], NYHA 3 [42.85% (group 2) vs. 61.34% (group 1)] and NYHA 4 [10.08% (group 2) vs. 25.21% (group 1)], p < 0.05.

Conclusions: Obtaining a more optimal heart rate with ivabradine in patients with congestive heart failure is reflected in an improvement in NYHA classification.

## Introduction

Introduction: Approximately 1-2% of the health care budget is consumed by heart failure patients, and the number of patients afflicted with heart failure appears to increase day by day [1]. Heart failure is a complex disease, resulting from either structural or functional deficits that adversely affect myocardial function, compromising our physiological circulation [1].

Despite multidrug therapy, these patients present with exacerbation of their disease, resulting in frequent admissions/readmissions with an increasing number of deaths [2]. According to a report, the readmission rate from three months to one year is 30-50%, in patients with heart failure [3].

Among all other factors, an increase in heart rate is a significant predictor of cardiovascular mortality and hospitalizations in patients with cardiac failure [4]. A resting heart rate of more than 60 directly increases the risk. Furthermore, a heart rate of 70 bpm is associated with a 34% increase in cardiovascular deaths and a 53% increase in hospitalizations in heart failure patients as compared to a heart rate less than 70 bpm [5].

Due to extensive evaluation of the role of heart rate in cardiac failure, a therapeutic agent, with a sole effect on heart rate is highly demanded. Ivabradine is a novel agent that significantly reduces heart rate without additional unwanted effects. Ivabradine inhibits the Ionic If current (Funny Channels) that modulates the pacemaker activity of the SA Node, with no additional effects on other ionic channels or receptors in the heart or vascular system, hence in patients with impaired left ventricular systolic function, the myocardial activity or intra-cardiac conduction is not affected. Ivabradine is reported to improve New York Heart Association (NYHA) class by 28% in patients with left ventricular systolic dysfunction [6,7].

Ivabradine has received Food and Drug Administration (FDA) approval in recent times. It is indicated to reduce hospitalization in worsening HF patients with a left ventricular ejection fraction (LVEF) of 35% or less, in sinus rhythm with a resting heart rate of 70 beats per minute (bpm) or greater, and who are either receiving maximally tolerated doses of beta-blockers or have a contraindication to beta-blocker use.

Ivabradine lowers heart rate by acting selectively and specifically on the cardiac pacemaker current (If) that controls the spontaneous diastolic depolarization in the sinoatrial (SA) node and hence regulates the heart rate. Ivabradine specifically affects the SA node, having no effect on blood pressure, intracardiac conduction, myocardial contractility, or ventricular repolarization [8].

The data on the efficacy of ivabradine as an add-on therapy are scarce in our Pakistani population. We studied the effect of the addition of ivabradine as an add-on therapy to the conventional heart failure treatment, including beta-blockers in the reduction of hospitalization and also in the improvement in heart failure symptoms.

## Materials and methods

This randomized controlled trial was conducted at the Medical Teaching Institute, Khyber Teaching Hospital, Peshawar. The duration of the study was nine months from November 1, 2020, to May 31, 2021. The sample size was 119 patients in each group (total 238 patients, WHO software for sample size determination) using 28% proportion in one group and 46.2% proportion in the other group, with a 95% confidence level and 90% power of the study. Non-probability consecutive sampling technique was used for data collection. The study was conducted after approval from the hospital ethical committee. The patients aged between 30 and 65 years and of either gender and presenting to the emergency department with heart failure symptoms (NYHA classes 3 and 4), having a heart rate of 80 bpm or above and in sinus rhythm. Echocardiography revealed left ventricular systolic dysfunction with an ejection fraction of less than 40%.

Patients with a recent (two months) myocardial infarction, scheduled coronary revascularization, severe congenital and rheumatic valvular heart disease, stroke, or transient cerebral ischemia within the previous four weeks were excluded. The study sample also did not include patients with atrial or ventricular pacing, sick sinus syndrome, sinoatrial block, second and third-degree atrioventricular block, positive family history, or congenital long QT syndrome were also excluded. Comorbidities like moderate or severe liver disease, severe renal disease, or anemia were considered before inclusion. The patients were randomly assigned into two groups, one group was already on standard treatment, and the other group was given ivabradine (5 mg twice daily) in addition to standard therapy.

All patients were subjected to a detailed history, followed by complete routine examination and baseline investigations, with included an ECG (Cardiofax) and echocardiography (Siemens’ Acuson cv-70). The improvement in the symptoms was observed during their hospital stay before discharge. Patients were followed through telephone contact and regular visits by evaluation of their NYHA heart failure classification, heart rate (by 12-lead ECG), and blood pressure, assess potential improvements in their symptoms at two months.

All of the above-mentioned information, including demographic features, were recorded in a pre-designed performa. Strict exclusion criteria were followed to control confounders and bias in the study results.

Data were analyzed using Statistical Package for Social Sciences (SPSS) version 16.0. Mean + standard deviation were calculated for continuous variables like age. Frequency and percentages were calculated for qualitative variables like gender and efficacy. Efficacy was compared between two groups using the chi-square test and the P-value less than or equal to 0.05 was considered significant. Efficacy was also stratified among age and gender to see effect modifiers. Post-stratification was done through chi-square evaluation, keeping p-valuation as above.

## Results

A total of 238 patients were included in the study. The mean age was 58.05 ± 4.98 (30-65) years. Females were 92 (38.65%) and males were 146 (61.34%). Based on the treatment given, the patients were divided into two groups, group 1 included patients on traditional therapy and group 2 included patients on ivabradine in addition to traditional therapy. The number of patients in each group was 119 (Table 1).

**Table 1:** Baseline characteristics of patients

Variable	Mean	Range	n=238	Percentage
Age (years)	58.05 ± 4.9	30–65		
Gender				
Male			146	61.34%
Female			92	38.65%
Groups				
Group 1 (traditional therapy)			119	50%
Group 2 (ivabradine)			119	50%

Overall, the enrolled patients displayed a wide distribution of admission heart rate and the mean heart rate was 85.4± 4.8 beats per minute (bpm). Symptoms were quantified according to the NYHA classification. At presentation, approximately 144 (60.50%) patients had NYHA class 3 while 94 (39.49%) were in NYHA class 4 in total. History of the previous hospitalization was provided by 150 (63.02%) patients in both groups (Table 2).

**Table 2:** NYHA class and previous hospitalization at admission in CHF patients NYHA: New York Heart Association, CHF: congestive heart failure

History	Frequency (n=238)	Percentage
Heart Failure Classification (NYHA)		
NYHA class 3	144	60.50%
NYHA class 4	94	39.49%
Previous hospitalization		
Positive history	150	63.02%
Negative history	88	36.97%

In group 1, 73 (61.3%) patients presented with NYHA 3 while 46 (38.65%) were in NYHA 4. Similarly in group 2, NYHA 3 and NYHA 4 were revealed in 71 (59.6%) and 48 (40.3%), respectively (Table 3).

**Table 3:** NYHA class at presentation between two groups in CHF patients NYHA: New York Heart Association, CHF: congestive heart failure

Intervention/therapy	Shortness of breath			
	NYHA3		NYHA4	
	Frequency	Percentage	Frequency	Percentage
Group 1 (traditional) n=119	73	61.34%	46	38.65%
Group 2 (ivabradine) n=119	71	59.66%	48	40.33%

Gender distribution showed that 90 (61.64%) male patients were in NYHA class 3 while 56 (38.357%) patients were in NYHA class 4. Similarly, 54 (58.69%) female patients presented in NYHA 3 and 38 (41.30%) in NYHA 4 (Table 4).

**Table 4:** Gender distribution of NYHA class in CHF patients NYHA: New York Heart Association, CHF: congestive heart failure

NYHA class	Gender			
	Male (n=146)		Female (n=92)	
	Frequency	Percentage	Frequency	Percentage
NYHA class 3	90	61.64%	54	58.69%
NYHA class 4	56	38.35%	38	41.30%

Treatment regimens are shown in Table 5. 

**Table 5:** Different medications used in CHF Patients ACE: angiotensin-converting-enzyme, CHF: congestive heart failure

Medication	Frequency n=238	Percentage
Diuretics + beta blockers	56	23.52%
Diuretics + ACE inhibitors	38	15.96%
Diuretics + beta blockers + ACE inhibitors	80	33.61%
Diuretics + beta blockers + ACE inhibitors + nitrates	64	26.89%
Total	238	100%

All of the patients were followed during their hospital stay for any complications. The patients were discharged to home on standard heart failure treatment and those in group 2 were on standard therapy as well as on ivabradine as an add-on therapy. Patients were followed for two months to assess potential improvements in symptoms and quantification in their NYHA classification. About 238 patients were followed for symptomatic improvement.

The mean heart rate at follow-up was 82.9±3.3 bpm in group 1, while 75.54±2.2 bpm in group 2. The reduction in the severity of the symptoms assessed as NYHA class on two months follow-up was more in group 2 versus group 1, with NYHA 2 [47.05% (group 2) versus 13.44% (group 1)], NYHA 3 [42.85% (group 2) versus 61.34% (group 1)] and NYHA 4 [10.08% (group 2) versus 25.21% (group 1), p = 0.02 (Table 6).

**Table 6:** NYHA class at two months follow-up of CHF patients *P=0.05 NYHA: New York Heart Association, CHF: congestive heart failure

Therapy	Shortness of breath						P-value (chi-square test)
	NYHA class 2		NYHA class 3		NYHA class 4		
	Frequency	%	Frequency	%	Frequency	%	
Group 1 (traditional) n=119	16	13.44%	73	61.34%	30	25.21%	
Group 2 (ivabradine) n=119	56	47.05%	51	42.85%	12	10.08%	0.02*

Efficacy in group 2 was found in about 78 (65.54%) patients (Table 7).

**Table 7:** Frequency of efficacy in ivabradine group at two months follow-up of CHF patients CHF: congestive heart failure

Group 2 ivabradine therapy	Frequency (n=119)	Percentage
Efficacy		
Yes	78	65.54%
No	41	34.45%

Results showed that efficacy in the ivabradine group was significant as compared to group 1, where it was effective in 50 (42.01%) patients (p = 0.01) as shown in Table 8.

**Table 8:** Comparison of efficacy at two months follow-up of CHF patients *P=0.05 CHF: congestive heart failure

Therapy	Efficacy				Total	P-value (chi-square test)
	Yes		No			
	Frequency	%	Frequency	%		
Group 1 (traditional)	50	42.01%	69	57.98%	119	
Group 2 (ivabradine)	78	65.54%	41	34.45%	119	0.01*
Total	128	53.78%	110	46.21%	238	

Similarly, stratification with respect to age and gender is shown in Tables 9-10, respectively.

**Table 9:** Comparison of efficacy according to age at two months follow-up of CHF patients *P=0.05 CHF: congestive heart failure

Age groups		Efficacy				Total	P-value (chi-square test)
		Yes		No			
		Frequency	%	Frequency	%		
Age 45–55 years							
	Group 1 (traditional)	18	52.94%	16	47.05%	34	
	Group 2 (ivabradine)	20	66.66%	10	33.33%	30	0.05*
	Total	38	59.37%	26	40.62%	64	
Age 55–65 years							
	Group 1 (traditional)	48	57.14%	36	42.85%	84	
	Group 2 (ivabradine)	55	61.11%	35	38.88%	90	0.02*
	Total	103	59.19%	71	40.80%	174	

**Table 10:** Gender-wise comparison of efficacy at two months follow-up of CHF patients *P=0.05 CHF: congestive heart failure

Gender		Efficacy				Total	P-value (chi-square test)
		Yes		No			
		Frequency	%	Frequency	%		
Male							
	Group 1 (traditional)	42	56%	33	44%	75	
	Group 2 (ivabradine)	43	60.56%	28	39.43%	71	0.03*
	Total	85	58.21%	61	41.75%	146	
Female							
	Group 1 (traditional)	26	59.09%	18	40.9%	44	
	Group 2 (ivabradine)	30	62.5%	18	37.5%	48	0.02*
	Total	56	60.86%	36	39.13%	92	

**Figure 1:**
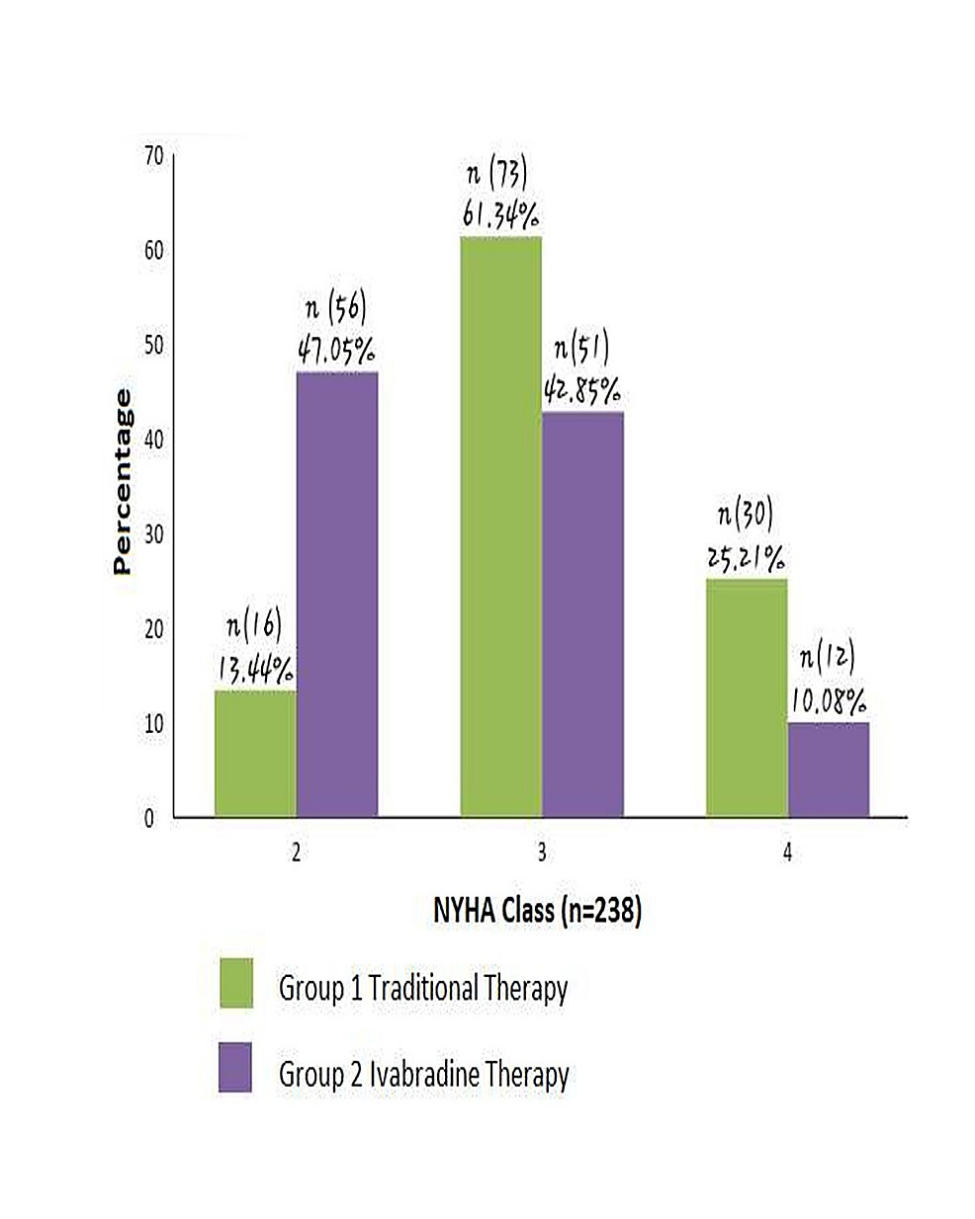
Comparison of NYHA class between two groups on two months follow-up NYHA: New York Heart Association

The improvement in the NYHA class on follow-up between two groups is shown in Figure 1.

## Discussion

Ivabradine has a dose-dependent reduction in heart rate. At present, the recommended dosages produce a reduction in heart rate of approximately 10 bpm whether the patient is at rest or exercising. The heart rate reduction has a linear effect with an increasing dose of ivabradine [9,10].

In our study, the mean age of the participants was 58.05±4.9 years, which was close to a sub-analysis of the SHIFT trial (60.7±11.3 years of age). The patients with severe symptoms of heart failure quantified by NYHA classification were included. Most of the patients were in NYHA 3 (60.50%) and NYHA 4 (39.49%) [11]. Similarly, in the sub-analysis of SHIFT, patients were in NYHA class 2 (58%) with remaining in class 3/4 (42%). The mean heart rate in our study at presentation was 85.4± 4.8 bpm, whereas in the SHIFT trial it was 79.8±9.0 bpm. As in our study, most of the patients were receiving recommended traditional HF therapies, including angiotensin-converting enzyme inhibitors and/or angiotensin II receptor blockers (95%) and a beta-blocker (90%) [12]. At two months of follow-up in the ivabradine treated group, the mean was 75.54 bpm with a reduction of 10 bpm, while in the sub-analysis it occurred 14.8, which was at 12 months of ivabradine supplementation.

There was an improvement of NYHA classification of approximately 18.49% for NYHA class 3, which was similar to the SHIFT sub-analysis of 24.2%; however, there was an improvement of approximately 15.13% in the case of NYHA 4. Moreover, there were 47.05% patients in NYHA 2 after ivabradine use, which demonstrated an improvement in NYHA classification because there were no patients in NYHA 2 who were included at enrollment of our study. In the SHIFT trial, there was a reduction of 16% (NYHA 3/4) in very severe in advanced NYHA classifications, which was similar to our study and 18% in the less severe group (NYHA 2) [13].

In another study, there was no reduction in the first-time readmission rate, but there was a significant reduction in the second-time readmission [14]. Similarly, in our study, the rate of readmission, in the beginning, was 61.5%, which was reduced significantly after two months of follow-up in the ivabradine group at 37.81%.

In an analysis performed in diabetic patients and ivabradine use, there was a significant reduction in primary endpoints, such as hospitalization, which again supported the use of ivabradine [14]. Ivabradine can prevent about 14 patient-years on average per year of follow-up as shown in a study done to estimate NNT (number needed to treat) [15].

In spite of all these promising results, the BEAUTIFUL (morbidity-mortality evaluation of the I f inhibitor ivabradine in patients with coronary disease and left-ventricULar dysfunction) heart failure patient trial concluded that there was no significant reduction in cardiac outcomes with ivabradine in patients with stable coronary artery disease and left ventricular dysfunction [15]. However, a reduction in the admission rate to be hospitalized for fatal and non-fatal myocardial infarction and coronary revascularization was found.

These differences between our study and other international studies are due to the fact that in the SHIFT trial there was a long follow-up interval and patients were assessed repeatedly at 28 days, 3 months, 6 months, and thereafter at one to two years. Moreover, the SHIFT trial was a multicenter study, whereas our study involved a single-center. Most of the complications related to ivabradine cannot be assessed properly in our study because of a short period of follow-up.

Limitations

There were certain study limitations. This was a single-center study where follow-up was limited to 60 days, so the ability of ivabradine in reduction of NYHA class beyond this time point could not be assessed. The study sample consists solely of patients with chronic heart failure and without any complications. Whether ivabradine can cause complications like myocardial infarction or complete heart block would have been of great help in utilizing data for its use in chronic heart failure patients with complications. Also, most of the patients were from a low socioeconomic class; therefore, the study findings cannot be generalized to a regional population. In addition, the number of patients enrolled in the study was small, therefore larger-scale studies are needed to validate the study findings.

## Conclusions

Increased heart rate has an important effect on outcomes in heart failure patients. Ivabradine, a pure heart rate, reducing age, can be used safely in heart failure patients, especially in those who are intolerant to the use of beta-blockers. Ivabradine may play an important role in reducing NYHA classification symptoms and is effective as an add-on therapy to the traditionally used medication for the treatment of systolic dysfunction.
